# Valvular Heart Disease in Pregnancy

**DOI:** 10.14797/mdcvj.1323

**Published:** 2024-03-14

**Authors:** Hilary Shapiro, Laith Alshawabkeh

**Affiliations:** 1 University of California, San Diego, San Diego, California, US

**Keywords:** pregnancy, valvular heart disease

## Abstract

Valvular heart disease is a common cause of peripartum cardiovascular morbidity and mortality. The hemodynamic changes of pregnancy and their impact on preexisting valvular lesions are described in this paper. Tools for calculation of maternal and fetal risk during pregnancy are also discussed. The pathophysiology and management of valvular lesions, both obstructive and regurgitant, are then described, followed by discussion of mechanical and bioprosthetic valve complications during pregnancy.

## Overview

The Centers for Disease Control and Prevention (CDC) estimates that nearly 30% of pregnancy-related deaths in the United States can be attributed to cardiovascular disease.^1^ With improvements in therapies to treat congenital heart disease (CHD) in infancy and childhood, more and more women with CHD are now reaching childbearing age and becoming pregnant, thus changing the epidemiology of chronic heart disease in pregnancy.^2,3^ Management of cardiovascular disease during pregnancy is important for clinical cardiologists because the normal hemodynamic shifts that occur in pregnancy can exacerbate existing cardiovascular issues and pose significant management challenges for the clinician.^4,5^ In this review, we describe the normal hemodynamic changes of pregnancy, their effects on the most common valvular abnormalities, and current management strategies.

## Hemodynamics of Pregnancy

Pregnancy causes significant cardiovascular shifts for the expecting mother. Throughout gestation, the patient will experience an increase in blood volume and cardiac output and a reduction in systemic vascular resistance (Table 1).^6,7^ Cardiac output (CO) increases early in pregnancy by 30% to 50% above prepregnancy levels and then remains elevated throughout pregnancy.^8^ This change is driven mostly by an increase in stroke volume and later by heart rate (HR) increases. The rate of CO increase plateaus in the second and third trimesters.^9^ A slow decrease in systemic vascular resistance (SVR) begins early in pregnancy. As the placental circulation matures, the SVR continues to drop until it reaches a nadir in the second trimester, reaching around 30% to 50% of preconception SVR levels; maternal SVR then gradually increases again towards the end of pregnancy.^10,11^ Plasma volume steadily increases throughout pregnancy.^5^ In total, these changes result in an increase of blood flow across cardiac valves and can worsen preexisting valvular gradients.

**Table 1:** Hemodynamic changes of pregnancy, labor, and delivery and the early postpartum period. CO: cardiac output; HR: heart rate: SVR: systemic vascular resistance


HEMODYNAMIC CHANGES OF PREGNANCY

Increased heart rate

Increased plasma volume

Decreased systemic vascular resistance

Increased cardiac output

Increased hypercoagulable state

**HEMODYNAMIC CHANGES OF LABOR AND DELIVERY**

Initial rapid increase in HR, stroke volume, CO

Return of blood from placenta to mother with uterine contractions (≤ 500 cc/ contraction), increases preload

**HEMODYNAMIC CHANGES OF EARLY POSTPARTUM**

CO peaks, ~80% higher than pre-labor CO

Increased preload

Normalization of SVR to prepregnancy levels (increased afterload)

Hypercoagulable state persists to ~8-12 weeks postpartum


Of particular importance for patients with mechanical valves is that pregnancy is a hypercoagulable state, which increases the risk of valve thrombosis.^12^ Further, after about 20 weeks of gestation, the gravid uterus is large enough to exert pressure on the inferior vena cava and cause partial obstruction and slowed venous return, which can also increase the risk of thrombosis and increasing valve gradients.^5^

## Hemodynamics of Labor, Delivery, and the Postpartum Period

Maternal hemodynamics during labor and delivery are complex, change quickly, and can be impacted by factors such as pain, delivery method, and choice of anesthesia and analgesia.^4^ Patients who are already minimally compensated may have trouble handling these abrupt changes. Cardiac output increases up to 30% during labor and up to 80% by 24 hours postpartum.^13,14^ Stroke volume and HR increase with the onset of labor and uterine contractions, which both increase CO even further; the changes to CO are greatest in the second stage of labor.^13,15^ Blood pressure transiently increases with each uterine contraction.^13^ Additionally, blood is returned from the placenta to the systemic circulation with each uterine contraction—an “auto transfusion” that can be as much as 500 ccs of blood at a time.^16^ Anesthesia and analgesia can blunt some of the HR and CO changes, but these medications can also cause hypotension and venous and arterial dilation.^5^ Finally, while blood loss is expected during both vaginal and cesarian delivery, the risk for hemodynamically significant blood loss and hypovolemia is generally greater with a surgical delivery.

Hemodynamic changes continue in the immediate postpartum period, and the 24 to 72 hours postpartum pose the highest risk for a patient to develop symptomatic heart failure from valvular lesions.^4^ As mentioned, CO is as much as 80% higher than prelabor CO within the first hours after delivery.^13^ Increases in preload following delivery and relief of inferior vena cava compression are accompanied by increases in SVR to prepregnancy levels. All of these changes can cause pulmonary congestion and the development of symptomatic heart failure.^5^ Finally, women are at the highest risk of thrombosis in the 6 weeks after delivery, and this risk does not return to prepregnancy levels until about 12 weeks postpartum.^12^

## Risk Stratification

With these changes in mind, it is important to properly risk stratify patients with valvular heart disease who are pregnant or considering pregnancy to better manage issues during pregnancy, discuss surveillance intervals, and formulate a delivery plan well in advance of the onset of labor.

There are three risk models that are often used to assess CV risk in pregnancy: the CARPREG II (CARdiac disease in PREGnancy), ZAHARA (Zwangerschap bij Aangeboren HARtAfwijkingen), and mWHO (modified World Health Organization) risk calculators. More recently, the DEVI (Adverse Cardiac Events in Valvular Rheumatic Heart Disease in Pregnancy) score was developed.

In the mWHO model, congenital and acquired heart lesions are categorized into four groups (I-IV) based on their risk to mother and fetus. Small, simple lesions or successfully repaired cardiac lesions are mWHO class I and pose minimal increased risk of mortality to mother. Severe mitral stenosis and severe symptomatic aortic stenosis are considered mWHO class IV lesions, meaning that pregnancy poses a severe threat to maternal life and is contraindicated.^17^ The mWHO classification was developed by expert consensus but was validated in the Registry of Pregnancy and Cardiac Disease study.^18^

The CARPREG II risk index is based on analysis from a large cohort of Canadian women with heart disease and CHD. It calculates the risk of complications based on a weighted point model: 3 points are given for mechanical valves, severe heart failure (New York Heart Association class III or IV symptoms), cyanosis, or prior cardiac events or arrhythmias; 2 points are assigned for high-risk valvular disease or left ventricular outflow tract obstruction, high-risk aortopathies, systemic ventricle dysfunction, pulmonary hypertension, or coronary artery disease; and 1 point is given for late pregnancy assessment or no prior cardiac interventions. Based on the patient’s total score, they are assigned a percent risk of CV complications during pregnancy, where more than 4 points is a 41% risk, 4 points is 22%, 3 points is 15%, 2 points is 10%, and 0-1 point is 5%.^19^

The ZAHARA II risk score was developed and validated in a large cohort of women with CHD. The score is based on assigned weighted points: mechanical valve prosthesis (4.25 points), left heart obstruction (2.5 points), history of arrhythmias (1.5 points), cardiac medications prior to pregnancy (1.5 points), cyanotic heart disease (1 point), systemic AV valve regurgitation (0.75 point), pulmonary AV valve regurgitation (0.75 point), and New York Heart Association functional class III-IV (0.75 point). A total score is then used to predict the risk of cardiac complications during pregnancy: A score > 3.51 (70% risk), 2.51 to 3.50 (43.1%), 1.51 to 2.50 (17.5%), 0.51 to 1.50 (7.5%), and 0 to 0.50 (2.9%).^20^

The DEVI score was recently developed to define risk in women with rheumatic heart disease during pregnancy. It was developed and validated in a low- and middle-income patient population. The score assigns points based on prior cardiac events, severity of mitral stenosis, pulmonary hypertension, presence of a prosthetic valve, and need for cardiac medications to arrive at a score between zero and 12. A recent validation study showed good discriminatory power and clinical utility in a cohort of Indian patients, in whom rheumatic heart disease is relatively common.^21^

A recent comparison study determined that the mWHO model is most powerful in predicting CV outcomes based on CV conditions and is a widely used tool for cardiac risk assessment in this patient population. However, each calculator provides a perspective on CV risk to the fetus and mother, and the recent development of the DEVI score highlights the importance of using calculators that address risk of common cardiac conditions in specific populations.^21,22^

In clinical practice, we feel that a risk score should be used to identify high-risk patients, and then patient-specific clinical data should be used to further refine the risk of cardiovascular events throughout the pregnancy. In a normal pregnancy, the biomarker N-terminal pro-B-type natriuretic peptide (NT-proBNP) should remain within normal limits throughout all three trimesters; an elevation above 200 pg/mL is associated with an increased risk of heart failure or preeclampsia.^23^ In pregnant women with stable valvular disease, NT-proBNP levels are often elevated above normal limits but should remain stable throughout pregnancy. A new increase in NT-proBNP levels above a patient’s baseline can signify a clinical worsening.^23^ Other studies have proven a role for cardiopulmonary exercise testing to further risk stratify women with valvular heart disease who are pregnant or considering pregnancy; for example, a blunted HR response to exercise was found in a study of 83 women to be associated with increased risk of adverse pregnancy outcomes.^24^

## Preconception Counseling

Ideally, severe and symptomatic stenotic and regurgitant valve lesions should be replaced or repaired prior to conception to minimize the risk for decompensation and maternal or fetal complications during pregnancy. These recommendations can be guided by mWHO or another risk score assessment. In practice, however, this is not always possible because patients sometimes become pregnant before appropriate valve management has been completed.

There are many contraceptive options available in reproductive-age women with severe valvular lesions who wish to prevent or delay pregnancy. Estrogen-containing contraception methods should be avoided in women with atrial fibrillation, atrial flutter, or mechanical valves due to the prothrombotic nature of estrogen.^4^ Long-acting reversible contraceptive methods that are progestin-only, such as an intrauterine device or subcutaneous implant, are extremely effective at preventing pregnancy and are well tolerated in cardiac patients.^25,26^

## Obstructive Lesions

Left-sided obstructive lesions are poorly tolerated during pregnancy (Table 2). These lesions are dependent on a narrow fluid and hemodynamic balance, where preload is required for forward flow, but any excess fluid can quickly progress to pulmonary edema and decompensation. In pregnancy, as CO increases, so too do the gradients across these narrow valves; therefore, close titration of diuretics and beta-blockers is required throughout pregnancy.^27^ Right-sided stenotic lesions are generally better tolerated, although risks remain to mother and fetus.

**Table 2:** Considerations for valvular lesions during pregnancy, by type. CO: cardiac output: TTE: transthoracic echocardiogram; AV: atrioventricular


**Obstructive Lesions**	Severe obstructive lesions are poorly tolerated in pregnancy

Aortic stenosis	Risk of decompensated heart failure throughout pregnancy with increased CO and plasma volume

	Follow with TTE each trimester

	Treat based on symptoms: consider AV nodal blocking agents, diuretics

Mitral stenosis	Risk of decompensated heart failure and atrial arrhythmias

	Follow with TTE each trimester

	Treat with AV nodal blocking agents, diuretics, consider anticoagulation for atrial arrhythmias

Pulmonary and tricuspid stenosis	Less common, generally better tolerated than left sided lesions

	Treat based on symptoms: consider AV nodal blocking agents, diuretics

**Regurgitant lesions**	Regurgitant lesions are generally better tolerated than obstructive lesions

	Treat based on symptoms: consider AV nodal blocking agents, diuretics, vasodilators

**Prosthetic valves**	Mechanical valves: obligate anticoagulation during pregnancy

	Bioprosthetic valves: valve degeneration may require repeat intervention during pregnancy


### Aortic Stenosis

The most common reason for aortic stenosis (AS) in reproductive-age women is a congenital bicuspid aortic valve. Women with a bicuspid aortic valve should be screened for an associated aortic coarctation or aortopathy.^28^ The risk of CV decompensation from AS increases throughout the second and third trimester as CO and plasma volume increase. These risks remain elevated during labor, delivery, and the subsequent 72 hours postpartum, when a large volume of blood is returned to the maternal circulation and while CO remains high.^10,13,16^ According to a cohort trial from the Pregnancy and Heart Disease Research Program at the University of Toronto, which followed 49 pregnancies in women with congenital aortic stenosis between 1986 and 2000, the risk of actual mortality to mother or fetus is extremely low (< 1%) even with severe AS, but the rate of morbidity and risks of complications during pregnancy increase in incidence with stenosis severity.^29^ Fetuses are at risk of intrauterine growth restriction, preterm birth, low birth weight, and respiratory distress. Throughout pregnancy, mothers have a roughly 15% risk of developing symptomatic heart failure and about 30% risk of arrhythmias according to data from a national Dutch registry study.^30^

During pregnancy, experts recommend a transthoracic echocardiogram (TTE) each trimester (with the final TTE occurring around 32 weeks at the peak of hemodynamic changes of pregnancy).^4^ Women diagnosed with bicuspid aortic valve should also have aortic imaging to assess for aorta dilation or coarctation.^31^

Heart rate optimization with AV nodal blocking agents such as metoprolol, propranolol, and diltiazem are safe in pregnancy and also help to optimize valvular gradients.^32^ Loop diuretics such as furosemide are appropriate to use in hypervolemic patients and those with pulmonary congestion, but care should be taken to not over-diurese the patient since this can cause hypotension in the mother (which can worsen preload-dependent AS).^5^ Severe symptomatic valvular disease unresponsive to medications can be treated with balloon valvuloplasty, though the timing of catheterization should target the second trimester if possible—after the completion of organogenesis—for the fetus to minimize the risks of radiation.^29,33,34^ These interventions should be considered as a last resort due to the risks to both mother and fetus; ie, cardiac surgery for the mother with cardiopulmonary bypass carries up to a 30% risk of fetal mortality.^35,36^ Ideally, women with severe AS who are contemplating pregnancy should be referred for surgery prior to conception.^37^

Vaginal delivery is possible for mild or moderate AS, though care should be taken to avoid hypotension with anesthesia. Cesarian section may be required for women with severe AS, but such decisions should be multidisciplinary and tailored to the patient.^38^ Further considerations regarding labor and delivery are described later in the article.

### Mitral Stenosis

Mitral stenosis (MS) is poorly tolerated by the pregnant patient. Pregnancy-related increases in HR cause shortened diastolic filling times, resulting in increases in left atrial pressures, pulmonary hypertension, and pulmonary congestion.^4^ Much as with AS, the mortality rates for mothers remain low, but the risks to the fetus are significant and increase with the severity of MS; for example, the fetus is at risk of growth restriction, premature birth, and low birth weight, according to two patient registries from Canada and Poland.^39,40^ Atrial arrhythmias (both atrial fibrillation and supraventricular tachycardias) are commonly seen in the pregnant patient.^41^

Recommended surveillance for mitral stenosis is similar to that of aortic stenosis, for which experts recommend a TTE each trimester, with the final TTE occurring around 32 weeks.^4^

The mainstay of medical therapy for MS in pregnancy involves (1) nodal blockers to extend the diastolic filling time and control atrial arrhythmias, and (2) diuretics to maintain euvolemia.^5,32^ Women with atrial fibrillation, left atrial appendage thromboses, or a history of embolic stroke should be given anticoagulation.^31^ Patients with very severe symptoms refractory to medical management can be referred for percutaneous balloon mitral valvuloplasty or even surgical replacement, although as with procedural management of AS, both options should be done only as a last resort after failed trials of medications.^42,43^

Vaginal delivery is possible in the large majority of MS patients, with the Cesarian section being reserved for symptomatic severe MS or for those with refractory pulmonary hypertension.^5^

### Pulmonary Stenosis

Pulmonary stenosis is generally very well tolerated in pregnancy, even when the obstruction is severe, and treatment should focus on symptom control.^44^ Some research indicates an increased risk of hypertension, pre-eclampsia, and RV dysfunction as a result of pulmonary stenosis in pregnancy.^45^ Valvotomy is possible in patients with symptoms of severe stenosis refractory to medical therapy, though this is needed in a very small number of patients.^46^

### Tricuspid Stenosis

Tricuspid stenosis is rarely seen in reproductive-age women and generally occurs in conjunction with other valvular lesions, CHD, or a degenerated bioprosthetic tricuspid valve. Medical therapy is directed at symptom control and HR control, with valvotomy or valve intervention reserved for refractory cases.^4,47^

## Regurgitant Lesions

Valvular regurgitation is generally better tolerated in pregnancy than valvular stenosis. As plasma volume and CO increase during pregnancy, so too does chamber dilation, which can result in larger regurgitant jets.^9,48^ However, these changes are often tolerated during pregnancy with less risk of complication than seen for stenotic lesions, especially in patients with preserved LV function. Women with concomitant systemic ventricular systolic dysfunction may be more prone to pulmonary congestion and symptoms from regurgitant lesions.^5^ Treatment of symptoms is focused on use of diuretics to maintain euvolemia, beta blockers for HR control, and vasodilators for afterload reduction. The use of renin-angiotensin-aldosterone system inhibitors is contraindicated in pregnancy due to their teratogenic effects. Hydralazine and nitrate combinations are often used during pregnancy instead. Labetalol, methyldopa, nifedipine, or amlodipine can also be used safely. Surgical intervention for regurgitant lesions is rarely required during pregnancy, except in cases of infective endocarditis complicated by valvular regurgitation due to the potential life-threatening risks to mother and fetus if it goes untreated.^49^

## Mechanical and Bioprosthetic Heart Valves

The clinician should pay close attention to the proper management of necessary anticoagulation in patients with mechanical heart valves. The hypercoagulable state of pregnancy, combined with the challenges of anticoagulation choices during pregnancy, make thromboembolic complications a serious risk for pregnant patients with mechanical valves.^50^

There are two different anticoagulation strategies that can be considered for mechanical valves in pregnancy. First is the use of warfarin since the risk of mechanical valve thrombosis is lowest with warfarin use; however, this medication crosses the placenta and carries teratogenic risks to the fetus at higher doses. Most guidelines recommend the use of warfarin for mechanical valve anticoagulation provided the patient requires no more than 5 mg/day to maintain a stable International Normalized Ratio level.^50^ Meta-analyses have shown that the risk of fetal toxicity is minimized below this level but increases quickly with doses above the 5 mg/day threshold.^51^

Low molecular weight heparin (LMWH) is an alternative anticoagulant option during pregnancy as it has excellent bioavailability and does not cross the placenta. LMWH should be dosed to anti-Xa goal 0.8 to 1.2 U/mL, with levels checked 4 to 6 hours after dose administration.^52^ LMWH is often administered via fixed weight-based dosing in the nonpregnant population, but the increase in glomerular filtration rate and therefore LMWH clearance as pregnancy advances places women at risk of subtherapeutic anticoagulation if dosing is not actively adjusted by monitoring anti-Xa levels.^50^ Unfortunately, studies have shown that patients are still at risk for thromboembolic complications, even if they maintain therapeutic anti-Xa levels.^53^

The choice of anticoagulant should be planned in a multidisciplinary fashion. Current guidelines recommend anticoagulation during pregnancy with warfarin ≤ 5 mg daily or LMWH in women who would require more than 5 mg of warfarin.^31,51,54^ Patients are generally transitioned from warfarin to unfractionated heparin before delivery, although there is still clinical equipoise on the appropriate timing for discontinuation of warfarin in favor of shorter-acting agents. The bleeding risk to both mother and fetus is high if labor begins while the mother is still anticoagulated with warfarin. Guidelines recommend low-dose aspirin for all prosthetic valves in the second and third trimesters.^31^

Bioprosthetic valves are generally well tolerated in pregnancy and have a significantly decreased risk of valve thrombosis compared with mechanical valves. Bioprosthetic valves also offer the benefit of avoiding the need for systemic anticoagulation, which, as mentioned, carries significant risks to the mother and fetus during pregnancy, although bioprosthetic valves do require daily aspirin. Compared with mechanical valves, however, bioprosthetic valves are less durable and often require repeat intervention. Studies have shown that up to 82% of women of childbearing age have structural valve disease 10 years after initial valve implant; this carries significance for pregnant women with CHD who received their initial valves multiple years prior to pregnancy. Observational data has noted that women with left-sided bioprosthetic structural valve disease are more likely to experience adverse events during pregnancy than women with right-sided bioprosthetic structural valve disease or women with normally functioning bioprosthetic valves.^55^ There is some observational data suggesting that valve degeneration accelerates during pregnancy. However this has not been validated in subsequent studies. It is possible that this valve degeneration noted during pregnancy merely represents the expected natural history of bioprosthetic valves.^56^

As previously mentioned, gradients across all valves increase during the hemodynamic changes of pregnancy, but this does not necessarily signify a de novo prosthetic valve lesion. It is important to directly assess valve morphology on echocardiogram to make a diagnosis of prosthetic valve degeneration. In a normal prosthetic valve, the elevated gradients of pregnancy should return to normal after delivery.

## Prepregnancy Counseling for Women with Heart Disease

Women of childbearing age desiring pregnancy and who have significant valve disease that necessitates surgical intervention should be carefully counseled by a multidisciplinary team with knowledge and experience in the management of heart disease in pregnancy. Experienced centers can offer surgical options that may not exist at lower-volume centers. For example, women with severe symptomatic AS were traditionally offered valve replacement with a mechanical or bioprosthetic valve. Other approaches such as the Ross operation or newer valve reconstruction techniques such as the Ozaki procedure can be discussed at specialty centers. Ultimately, the choice of the operation should be weighed in a multidisciplinary fashion, engaging the patient as the final arbiter of these options after fully understanding the immediate, peripartum, and long-term risks of all options.

## Arrhythmias

Arrhythmias are a common complication of valvular lesions in both pregnant and nonpregnant patients. Their management is discussed in greater detail elsewhere in this journal, but briefly, nodal blockers, are safe in pregnancy, as are adenosine, flecainide, and sotalol. Synced direct current cardioversion is safe in pregnancy but is generally reserved for unstable patients or those who have failed medication trials.^57^ Anticoagulation principles are the same as those for mechanical mitral valves.

## Delivery Planning, Labor, and Delivery

A multidisciplinary cardio-obstetrics team is beneficial in the management of pregnant patients with valvular disease and also in the planning for labor and delivery.^58,59,60^ Overall, women with regurgitant valvular lesions generally tolerate the hemodynamic shifts associated with labor and delivery. Women with stenotic lesions often require close cardiac monitoring during this period.^4^

Early use of regional anesthesia with an epidural anesthetic is recommended to minimize pain and hypertension.^15^ For low-risk patients (women with minimal symptoms or limitations in functional status), a spontaneous vaginal delivery is appropriate, although the obstetric provider should consider an assisted second stage of labor to limit periods of prolonged Valsalva during pushing.^5^ A scheduled Cesarian section is recommended for women with severe valvular lesions or symptoms.^38,61^ Cardiac anesthesia specialists may be required for high-risk cases, and this decision should ideally be made by the cardio-obstetrics team in advance of the anticipated delivery day to allow for proper staffing and planning. Endocarditis prophylaxis is generally not necessary.^38^

Anticoagulation management should also be addressed ahead of delivery to minimize the risks of epidural-associated hemorrhage, postpartum hemorrhage, and neonatal intracranial hemorrhage.^62^ As mentioned, expert opinion recommends discontinuation of warfarin at 36 weeks and switching patients to LMWH or unfractionated heparin for the remainder of pregnancy. Anticoagulation should be held the day prior to anticipated delivery.^63^ Unfractionated heparin can be restarted 6 hours after vaginal delivery or 12 hours after Cesarian section in uncomplicated deliveries without ongoing excessive bleeding.^64^ After delivery and discharge, women previously on warfarin may resume it since it is not passed through breastmilk and is therefore safe to use during and after the postpartum period.^63^

## Conclusion

The hemodynamic changes of pregnancy pose unique management challenges for the clinician. Regurgitant lesions are generally better tolerated than stenotic ones, though severe valvular abnormalities of either type can cause significant symptoms and functional limitations to mother and grave threats to the fetus. The mWHO model is the most well-used in assessing the pre-conception risk of CV events during pregnancy, and pre-conception planning is often required to exchange prepregnancy teratogenic medications for ones that are nonteratogenic. In women who have severe symptoms despite maximal medical therapy, percutaneous and surgical valvular interventions can be considered, although these should generally be last-resort therapies as the risks of fetal mortality are high. Spontaneous vaginal delivery is generally safe in the majority of patients, but scheduled Cesarean-section delivery with cardiac anesthesia is appropriate for higher-risk patients. Anticoagulation strategies require advance planning and close monitoring. As advances in cardiovascular care allow more women with congenital valvular lesions, rheumatic heart disease, and prosthetic valves to reach childbearing age, cardiology providers can expect to see more of these patients in their clinics and hospitals.

## Key Points

- Valvular heart disease is a significant cause of pregnancy-related morbidity and mortality. Globally, the majority of valvular lesions in women of childbearing age are due to rheumatic heart disease; in developed countries, prior congenital heart disease is now the most common reason for valvular heart disease in pregnancy.
- Left-sided obstructive lesions are less tolerated than regurgitant and right-sided valvular lesions. Early identification of women at high risk facilitates establishing a commensurate surveillance strategy and managing the changes in intravascular volume, heart rate, and blood pressure of pregnancy, all of which can compound valvular lesions.
- Prosthetic valves pose unique challenges for the cardio-obstetrics provider: knowledge of anticoagulation options and risks of valve degeneration and complications are important.
- Labor, delivery, and the early postpartum period are especially high-risk periods for pregnant women with valvular heart disease. Careful multidisciplinary delivery planning and close postpartum follow-up are advised.
